# The Trochlear Bisector as a New Landmark for Kinematic Alignment in Total Knee Arthroplasty: A Radiographic Study

**DOI:** 10.3390/jcm13123548

**Published:** 2024-06-17

**Authors:** Francesco Iacono, Tommaso Bonanzinga, Berardo Di Matteo, Alberto Iacomella, Michelangelo Delmedico, Francesco Manlio Gambaro, Alberto Favaro, Maurilio Marcacci

**Affiliations:** 1 Department of Biomedical Sciences, Humanitas University, Via Rita Levi Montalcini 4, Pieve Emanuele, 20072 Milan, Italy; francesco.iacono@humanitas.it (F.I.); tommaso.bonanzinga@hunimed.eu (T.B.); berardo.di_matteo@humanitas.it (B.D.M.); alberto.iacomella@humanitas.it (A.I.); mdelmedico@asst-pg23.it (M.D.); francesco.gambaro@humanitas.it (F.M.G.); maurilio.marcacci@humanitas.it (M.M.); 2 IRCCS Humanitas Research Hospital, Via Manzoni 56, Rozzano, 20089 Milan, Italy; 3 Department of Traumatology, Orthopaedics and Disaster Surgery, Sechenov University, 119991 Moscow, Russia; 4 ASST Papa Giovanni XXIII di Bergamo, 24127 Bergamo, Italy

**Keywords:** knee, alignment, radiographs, total knee arthroplasty, trochlear groove

## Abstract

**Background**: In recent years, there has been considerable interest in prosthetic alignment techniques for total knee arthroplasty (TKA), particularly in the so-called kinematic alignment, which aims to restore the knee’s native alignment. However, implementing this technique requires specialized instruments and procedural steps that can be laborious. This study introduces the bisector of the trochlear groove as a reliable landmark for performing the distal femoral cut while maintaining parallelism with the native femoral joint line. **Methods**: Three orthopedic specialists assessed 110 X-ray images of full-leg, weight-bearing lower limbs obtained from healthy individuals between January 2021 and December 2022. The bisector of the trochlear groove was identified on the X-ray images, and the angle between this bisector and the femoral joint line was measured. The consistency of these measurements across repeated assessments and different examiners was evaluated. **Results**: The bisector of the trochlear groove was found to be perpendicular to the femoral joint line, with a mean angle of 89.4°. The inter-rater reliability was 68% within ±1.3° from the mean, while the intra-rater reliability was 82% within ±1.5° from the mean. **Conclusions**: These results suggest that by performing a femoral cut perpendicular to the bisector of the trochlear groove, surgeons can inherently restore the femoral joint line of the native knee in patients where the native joint line is no longer identifiable due to the effect of osteoarthritis. This method may offer a viable and straightforward alternative to the standard surgical technique currently practiced for kinematic alignment in TKA.

## 1. Introduction

In total knee arthroplasty (TKA), orthopedic surgeons traditionally rely on mechanical alignment to position prosthetic components. This involves cutting the distal femur and proximal tibia perpendicularly to their respective mechanical axes, aiming for a neutral 180° hip-knee-ankle angle (HKA) to distribute the load evenly across the knee joint (i.e., between the medial and the lateral compartment) and, by doing so, minimize wear and loosening [1,2,3]. However, this mechanical alignment often differs from the natural alignment of the limb and knee as only a small percentage of subjects show a neutral native HKA axis [4,5], resulting in dissatisfaction in approximately 20% of patients who undergo TKA [6,7] and altering the physiological femoral and tibial joint lines. Alterations in the joint lines affect the described tibiofemoral and patella femoral kinematics of the natural knee, which typically occur around fixed axes [8,9,10,11,12]. Specifically, knee flexion and extension pivot around the cylindrical (or primary) femoral axis, which is anchored in the femur and defined by the circular profiles of the posterior condyles from 20° to 120°; tibial internal and external rotation occur around the longitudinal tibial axis, fixed within the tibia; and patella flexion and extension revolve around the secondary femoral axis, which is parallel, proximal, and anterior to the cylindrical femoral axis.

In an attempt to replicate the complexity of knee kinematics and improve the surgical outcomes, more recently, a new alignment approach has been introduced in TKA: kinematic alignment. Setting the joint line more anatomically than mechanically aligned TKA, kinematic alignment demonstrates better patient satisfaction, knee function, and flexion [13,14,15]. At the core of kinematic alignment lies the restoration of the patient’s pre-arthritic joint lines, and to achieve this, the femoral component should be aligned with the primary femoral axis both distally (full extension) and posteriorly (full flexion) [16]. However, the technique described by Howell for achieving kinematic alignment [13] requires dedicated caliper instruments and consists of several procedural steps that can be laborious to implement. Based on these premises, the objective of the present article is to describe the bisector of the trochlear groove as a new anatomical landmark for kinematically aligning the femoral component in TKA with the aim of streamlining the kinematic alignment procedure and reduce the sources of potential errors.

Our hypotheses are that the bisector of the trochlear groove is perpendicular to the femoral joint line and, consequently, perpendicular to the primary femoral axis. Therefore, by resecting the distal femur perpendicularly with respect to this bisector, it is possible to align the femoral component to the primary femoral axis, as proposed by kinematic alignment.

## 2. Materials and Methods

The dataset used in this study consisted of full lower limb X-rays in the anteroposterior (AP) view. Demographic and clinical characteristics of the patients included in the study are reported in Table 1. The data refer to healthy patients who underwent X-ray acquisition between January 2021 and December 2022 at our tertiary referral orthopedic institution. Acknowledging the heterogeneity between the contralateral legs of the same patients, attributed to leg dominance as supported by Gale et al. [17], this study treated the legs of the same patients as an independent entity. Written consent for anonymized data use for scientific purposes was obtained from all patients. Since the study solely involved radiographic analyses without clinical data and all imaging data were anonymized, no approval from an ethical committee board was sought. No supplementary radiographic projections were acquired. The AP view radiographs of the entire lower limbs were consistently captured with the knee fully extended and the patella oriented forward while ensuring the beam centered on the knee joint. Images were excluded if they displayed signs of osteoarthritis (OA) or post-traumatic deformities or if the patients had undergone previous surgical interventions, such as joint replacement, osteotomy, or anterior cruciate ligament reconstruction. Additionally, images were excluded if there was incorrect limb rotation during image acquisition, as evaluated by assessing the patellar position and the lesser trochanter profile.

**Figure 1:**
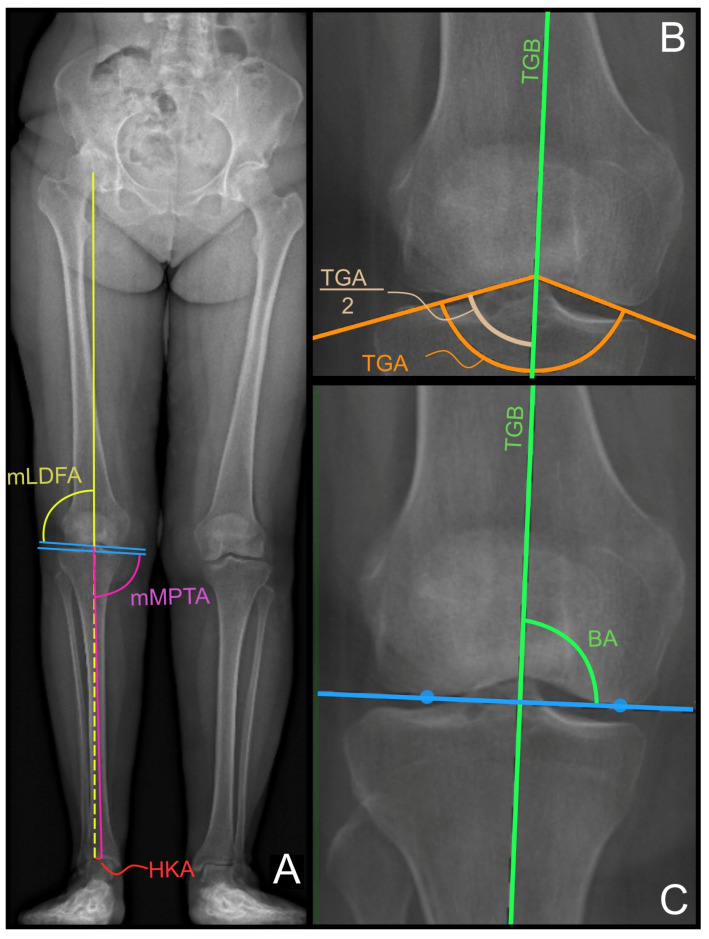
(**A**) The AP view of a full leg and weight-bearing acquisition of a healthy 31-year-old female. The femoral mechanical axis and the tibial mechanical axis are reported in the solid yellow and purple lines, respectively. The dashed yellow line represents the prolongation of the femoral mechanical axis. The HKA (in red), mLDFA (in yellow), and mMPTA (in purple) are measured as described in [18]. (**B**) A zoomed version of (**A**) on the right knee, with the representation of the TGA and TGB defined as per the presented technique. (**C**) The definition of the BA is the angle between the TGB and the femoral joint line.

Three members of our orthopedic unit with different levels of experience assessed the inter-observer reliability of the collected images: one senior orthopedic surgeon (F.I.), one junior orthopedic surgeon (M.D.), and one orthopedic resident (A.I.). Moreover, the same senior surgeon evaluated the same radiographic images at three different time points, each spaced two weeks apart, to evaluate the intra-rater reliability. Each investigator assessed the following parameters, defined in [18]: the hip-knee-ankle (HKA) angle, which was expressed as a deviation from 180° with a negative value for varus and positive value for valgus alignment; the mechanical lateral distal femoral angle (mLDFA) and the mechanical medial proximal tibial angle (mMPTA), as depicted in Figure 1A.

The bisector of the trochlear groove (TGB) was defined as follows:

1. Two lines are drawn tangentially to the medial and later aspects of the trochlear groove, starting from the radiographic apical midpoint of the inter-condylar groove. The trochlear groove angle (TGA) is measured as an angle subtended by these two lines (see Figure 1B, orange lines);
2. The TGB is drawn as the line that bisects the TGA and passes through the radiographic apical midpoint of the inter-condylar groove (see Figure 1B, green line);
3. The bisector angle (BA) is measured as the medial angle between the TGB and the distal femur joint line, which is defined as the line passing through the two most prominent points of the medial and lateral condyles (refer to Figure 1C, respectively, the green angle, blue line, and blue dots).

### Statistical Method

The statistical analysis was conducted using IBM SPSS version 25.0. Intra-rater and inter-rater reliability analyses were carried out utilizing the extended version of the Bland and Altman plot, and the intra-class correlation coefficient was calculated through two-way random effects, absolute agreement, and multiple measurements/raters. The extended Bland and Altman plot illustrates the difference from the mean against the mean of the raters (inter-raters) or the measurements (intra-raters) [19]. To assess the agreement of the continuous measurements among multiple observers, we employed the limits of agreement with the mean (LOAM) for multiple observers and multiple measurements per observer, as introduced by Jones et al. [20]. This method demonstrates how much an individual observer’s measurement can deviate from the mean measurement of all the observers. The inter-subject repeatability coefficient (RC) was determined based on the algorithm outlined by Bland and Altman in 1986 [21].

## 3. Results

The average measurements of the BA obtained by the three observers are detailed in Table 2, while the measurements recorded by the same observer at three distinct time points are presented in Table 3.

**Figure 2:**
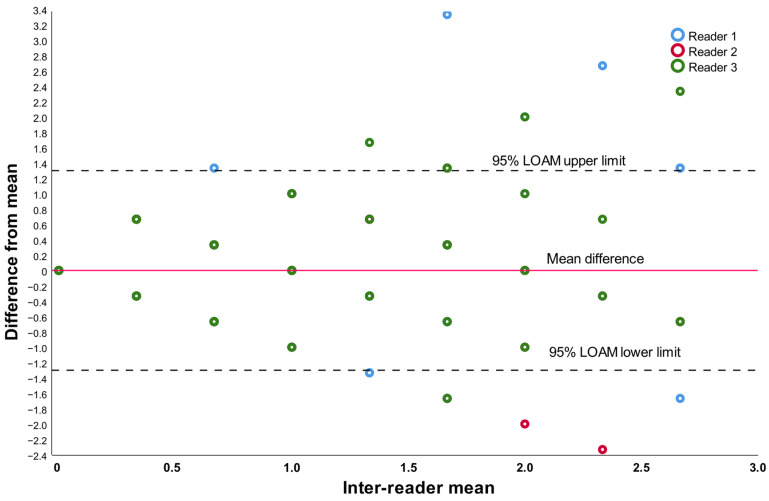
Extended Bland and Altman plot for the inter-observer agreement.

In relation to the additional knee alignment parameters, the average HKA value was 180° with a standard deviation of 2.48°, the mean mLDFA measurement was 87.6° with a standard deviation of 1.88°, and the mMPTA averaged 88° with a standard deviation of 2.2°. Concerning the inter-observer evaluation, the extended Bland and Altman plot indicated a limit of agreement of ±1.3°, while the intra-class correlation coefficient using a two-way random effects model was calculated at 0.677 (95% CI 0.556–0.769), as reported in Figure 2.

**Figure 3:**
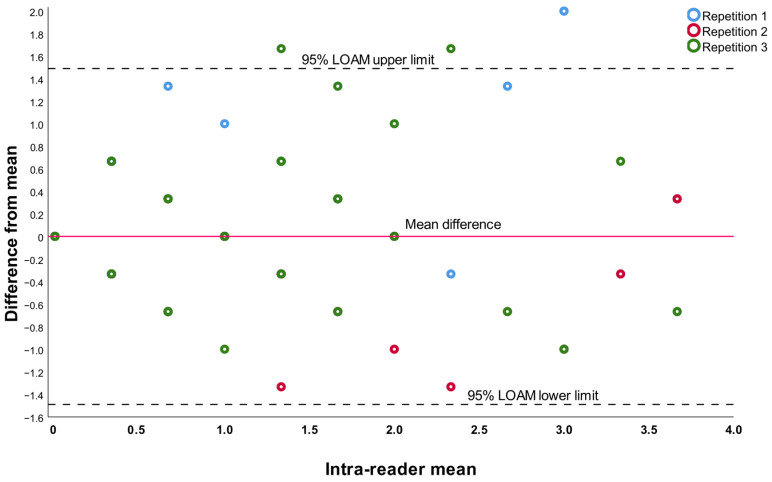
Extended Bland and Altman plot for the intra-observer agreement.

For the intra-observer analysis, the limit of agreement of the extended Bland and Altman plot was ±1.5°; the intra-class correlation at three time points with the two-way random effects model was 0.823 (95% CI 0.757–0.873), as shown in Figure 3.

The inter-subject repeatability was ±2.5°, higher than the limit of agreement of the inter- and intra-observer analyses.

## 4. Discussion

The primary discovery of the present work reveals that the newly explored anatomic landmark, i.e., the bisector of the trochlear groove, exhibits a perpendicular orientation to the femoral joint line in the anteroposterior weight-bearing radiographs of healthy lower extremities. The measurements showed moderate reproducibility. Specifically, an inter-reader agreement of 67.7% was established among the three observers, while an intra-reader agreement of 82.3% was achieved across temporally spaced assessments by the same observer. Notably, one of the three observers was a second-year resident, indicating a relatively inexperienced eye for X-ray interpretation; nevertheless, this observer demonstrated substantial concordance with the two senior observers. Furthermore, the inter-subject repeatability of ±2.5° exceeded the limits of agreement identified in the inter- and intra-observer analyses and remained relatively small in general terms, thereby indicating a low overall variability between the subjects. This suggests that the bisector of the trochlear groove not only serves as a promising novel landmark for knee alignment but also represents a readily reproducible and measurable parameter, demonstrating satisfactory intra- and inter-reader agreements as well as inter-subject repeatability.

The average values of the HKA, mLDFA, and mMPTA measurements aligned closely with the findings documented in the existing literature [18]. Given that the confidence interval for all three aforementioned measurements was within ±2°, the observed variability in limb alignment further supports the notion of substantial diversity in native knee alignment, as elucidated by Bellemans et al. [22].

Based on the findings of this study, the trochlear bisector could potentially inform the femoral distal cut in total knee arthroplasty following a kinematic alignment approach in individuals with OA. Indeed, the bisector of the trochlear groove taken at the level of the intercondylar notch is likely to remain unchanged over time, as it is generally not affected by OA, unless in the case of severe patellofemoral dysplastic arthritis or significant long-term axial deviations of the limb. This makes its bisector a potential landmark for restoring native pre-arthritic alignment during TKA procedures. By utilizing the coronal slope of the distal femoral resection determined using the trochlear bisector, implant alignment can be pre-operatively calculated using standard full weight-bearing radiographs, simplifying intra-operative procedures required by the original Howell’s technique without requiring specialized instruments. This easily identifiable and reproducible landmark has the potential to streamline implant positioning, potentially reducing operating times, minimizing anesthesia exposure, and enhancing surgical efficiency. Therefore, it could offer a straightforward alternative to Howell’s caliper resections for achieving kinematic alignment in everyday clinical practice.

We chose to use pre-operative X-ray radiographic images to measure this newly identified landmark, as they are widely available and commonly used in orthopedic practice.

Although the intercondylar notch has already been suggested as a useful landmark for determining resection depth in TKA because it is believed to be unaffected by pathological changes [23], to the best of our knowledge, the present study represents the first attempt to use its bisector for aligning the femoral distal cut in a TKA procedure.

It is important to acknowledge certain limitations of the current study. Firstly, in patients with severe OA, defining the bisector angle might be challenging due to the presence of osteophytes, potentially introducing bias in the bisector calculation. Intra-operative analyses are ongoing to assess whether this landmark remains intact in arthritic knees. Another limitation is the moderate inter-rater agreement observed, only 67.7% within ±1.3°. In future studies, incorporating CT scan-based measurements may enhance the reliability of the findings and offer stronger evidence supporting this novel anatomical landmark, although acquiring CT scans of healthy knees raises ethical concerns due to radiation exposure. Lastly, the literature highlights the low reliability of lower limb assessment on plain radiographs due to their two-dimensional nature. Every 5° change in the internal or external rotation of the lower limb is reported to correspond to a 0.2° modification in the HKA angle [24]. To address this limitation, we standardized the X-ray acquisition according to the guidelines described by Varatojo et al. [18].

At the moment, the bisector of the trochlear groove is used in our surgical practice to assist in determining the final varus-valgus alignment of the femoral component during TKA surgery. However, by including a CT-based study in pre-operative planning, it is possible to extend the application of this landmark to defining not only the coronal resection slope but also the axial rotation and flexion–extension angles of the femoral component. Furthermore, if the present alignment approach proves effective with rigorous scientific evidence and clinical validation, its application could be facilitated by robotic-assisted surgery. Robotic approaches have been shown to improve implant positioning accuracy based on precise pre-operative planning [25]. Future studies may also include computational simulations to investigate the biomechanical outcomes of this alignment technique, assessing load distribution on prosthetic components under different alignment scenarios, as demonstrated in previous studies [26,27].

## 5. Conclusions

The present study introduces a novel radiographic landmark, namely the bisector angle of the trochlear groove, which has the potential to streamline the attainment of kinematic alignment in TKA procedures. Our findings suggest that performing a femoral cut perpendicular to the bisector of the trochlear groove leads to:

- A natural mechanical lateral distal femoral angle (mLDFA);
- The positioning of the femoral component that meets the principles of kinematic alignment.

An ongoing in vivo surgical study, aims to validate the effectiveness of the bisector angle method in achieving kinematic alignment in TKA patients.

## Figures and Tables

**Table 1:** Demographic and clinical characteristics of patients.

Summary		Laterality		Sex		Age (y/o)		
X-rays	Patients	Unilateral	Bilateral	Male	Female	Min	Max	Mean
110	59	8	51	62.7% (37)	37.3% (22)	19	38	28.9

**Table 2:** Mean BA values obtained by the three observers. Legend: BA = bisector angle, SD = standard deviation.

	Observer 1	Observer 2	Observer 3
BA (SD)	89.5° (±1.25)	89.3 (±0.96)	89.2 (±1.1)

**Table 3:** BA values obtained at three different time points (spaced 2 weeks apart) by the senior observer. Legend: BA = bisector angle, SD = standard deviation.

	Observer 1-Time 1	Observer 1-Time 2	Observer 1-Time 3
BA (SD)	89.6° (±1)	89.5 (±0.96)	89.5 (±1.1)

## Data Availability

We are currently in the process of updating our database on Zenodo.
